# A Case Report of a Patient With Post-COVID-19 Dysphagia Who Required Antibiotics Following Intraoral Acupuncture

**DOI:** 10.7759/cureus.93251

**Published:** 2025-09-26

**Authors:** Yiu Ming Wong, Renato Mesias Calderon

**Affiliations:** 1 Physical Rehabilitation, Koumeido Shinkyuin Clinic, Taipei City, TWN

**Keywords:** acupuncture point, acupuncture therapy, acupuncture treatment, coronavirus disease 2019 (covid-19), post-covid-19 conditions

## Abstract

The purpose of this case report is to highlight an uncommon adverse event following a new form of acupuncture, intraoral acupuncture. An adult male patient who had been endotracheally intubated as part of COVID-19 treatment experienced swallowing difficulty. After being treated by a speech therapist for nine months, he started undergoing intraoral acupuncture for his dysphagia. Following this treatment, he developed local ulceration on his uvula and palatopharyngeal arches two days after the treatment. Due to a high suspicion of acupuncture-induced local infection, his physician gave a course of oral antibiotics for the intraoral ulcerations. The lesions completely healed 14 days after the ulcerations were initially observed. This case underscores the importance of vigilance and monitoring for adverse events in acupuncture practice.

## Introduction

Intraoral acupuncture is a new form of acupuncture therapy based on the theory of traditional Asian medicine, primarily involving invasively stimulating the tongue, uvula, and palatopharyngeal arches with acupuncture needles [1]. While it is not a standard or widely recognized practice, it has been used to relieve post-operative dental pain [2] and reduce excessive drooling (sialorrhea) [3], treat radiation-induced dry mouth (xerostomia) [4], and potentially help with obstructive sleep apnea [5] and neurosensory swallowing difficulty (dysphagia) [6]. Theoretically, the acupuncture needle insertions can stimulate sensory nerves in the mouth, which send afferent signals to the brain. In turn, this triggers the release of neurotransmitters such as serotonin and norepinephrine, which inhibit pain and promote healing [7]. However, because of its invasive nature, acupuncture carries a risk of infections if proper sterile techniques are not followed at the needling sites [8]. The present case report concerns an adult patient with post-COVID-19 dysphagia who developed oral ulcers and required antibiotics following intraoral acupuncture.

## Case presentation

A 39-year-old man was admitted to a public hospital in China for treatment of coronavirus disease 2019 (COVID-19) on August 21, 2023. The patient expressed self-perceived swallowing difficulty after the acute phase of COVID-19, during which he had required endotracheal intubation for five days. His post-COVID-19 dysphagia was confirmed with a modified water-swallowing test on October 15, 2023. The test employed a five-point scoring system; his score of two was deemed abnormal and indicative of a swallowing disorder, while scores of four or five are considered normal [9].

Dysphagia is one of the post-COVID-19 complications that ranges from mild discomfort in swallowing to life-threatening aspiration; the possible cause of the patient's dysphagia was mechanical laryngeal trauma from the endotracheal intubation, which resulted in edema of the vocal cords and arytenoids, leading to neurosensory partial paralysis of the vocal cords even after extubation [10-13]. Already on a soft diet, the patient experienced difficulty swallowing, with occasional choking and coughing during meals.

After one-on-one swallowing re-education provided by a speech therapist (twice a week for nine months between November 2023 and July 2024), the patient was not satisfied with the improvement in his swallowing issue. Then he turned to alternative medicine; after seeking treatment at a traditional Chinese medicine clinic in Hong Kong, the patient agreed to undergo intraoral acupuncture on September 12, 2024. During the intraoral acupuncture procedure, the patient sat in an inclined chair with his head tilted. The acupuncturist held the tongue with dry gauze using one hand and then quickly needled the intraoral acupoint areas with the needle held in the other hand [1]. In total, eight needlings were done: five insertions for the tongue, one insertion for the uvula, and two insertions for the bilateral palatopharyngeal arches. Two days after undergoing the intraoral acupuncture, he had a chief complaint of soreness in the posterior pharyngeal wall, and his swallowing difficulty worsened.

**Figure 1:**
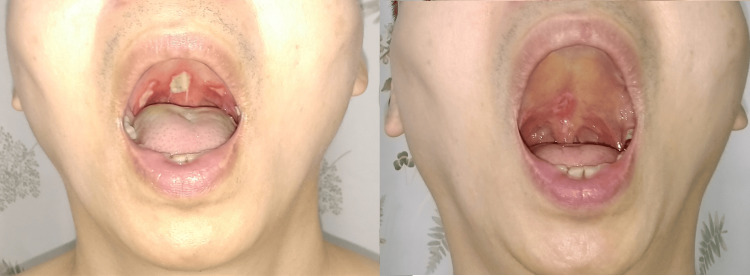
(Left) Ulcers were noted on the uvula and palatopharyngeal arches two days after intraoral acupuncture; (right) 14 days after the ulcerations had been first observed, the sores fully healed.

After visiting a medical doctor’s office on September 15, 2024, the physician revealed a 1 cm×0.7 cm ulcer within the base of the uvula, a 0.8 cm×0.5 cm ulcer medial to the right tonsil, and a 0.5 cm×0.3 cm ulcer medial to the left tonsil (Figure 1), while his oral hygiene was unremarkable. Also, no enlarged lymph node in the neck was palpable. The patient was a sedentary worker and had no other pre-existing long-term conditions requiring medication. His body mass index (BMI) was 23.2 kg/m², which was considered slightly overweight according to the WHO Asia-Pacific BMI classification (18.5-22.9 kg/m²) for Asian men.

Due to a high suspicion of acupuncture-induced local infection, antibiotics (Augmentin 375 mg, twice a day×five days) and a corticosteroid (Triamcinolone acetonide 0.1% dental paste, twice a day×seven days) were given for the intraoral ulceration. The lesions completely healed 14 days after the ulcerations were initially observed (Figure 1, right), and the patient reported that his swallowing difficulty returned to pre-adverse event status. Also, the patient decided to discontinue the acupuncture treatment.

## Discussion

The differential diagnosis for intraoral ulcers can be broad, including autoimmune diseases, nutritional deficiencies, oral cancer, systemic diseases, infections, or trauma [14,15]. The locations of the ulcers correspond with the acupuncture needle placements, suggesting that they were likely caused by needling-induced trauma, which disrupted the oral mucous membranes, allowing microorganisms to grow. Based on the patient’s statement, no pre-procedural mouthwash (e.g., antimicrobial mouthwash) was administered to him before the intraoral acupuncture. Using an antimicrobial mouthwash before the procedure may help reduce the risk of introducing bacteria or other pathogens to the needle insertion sites.

However, there is no confirmed explanation for the tongue, uvula, and palatopharyngeal arches that had been acupuncture-needled, but only the uvula and palatopharyngeal arches had ulceration afterwards. The branches of the external carotid artery supplied all three structures. However, the tongue relies primarily on the lingual artery, while the uvula and palatopharyngeal arches share supplies from the facial, maxillary, and pharyngeal arteries [16,17]. In comparison among the three structures, the tongue is a muscular organ with dense sensory innervation. In contrast, the uvula and palatopharyngeal arches are composed of soft tissue and are morphologically smaller. Therefore, the uvula and palatopharyngeal arches would sustain relatively more stress from a given identical invasive needling than the tongue.

Infectious adverse effects following acupuncture treatment are uncommon but can cause severe outcomes [8]. While previous cases typically occurred in the cutaneous tissues of the torso and limbs [18,19], the present case may be the first reported adverse event linked to intraoral acupuncture. Considering the balance between the proposed benefits (including improvement of post-operative dental pain [2], sialorrhea [3], and radiation-induced xerostomia [4]) and the risks of intraoral acupuncture, traditional invasive needling may not be the optimal approach for all patients, particularly pediatric patients. To minimize the risk of needle-induced puncture wounds, the choice of needle size should be as fine as possible, usually ranging from 0.12 to 0.18 mm. To further minimize complications associated with intraoral acupuncture, clinicians should consider using laser acupuncture as an alternative to traditional steel needles when it is necessary to stimulate intraoral acupuncture points. Laser acupuncture is a non-invasive technique that uses low-intensity laser irradiation (wavelengths commonly used between 635 and 830 nm), rather than traditional stainless-steel needles. Patients typically experience no or slight warmth during laser acupuncture [20].

## Conclusions

Intraoral acupuncture is not a mainstream practice even in Asia, where it originated, and also not an evidence-based medicine yet. Although suggested as a potential treatment for neurosensory swallowing and drooling issues, it is essential to approach this therapy with caution due to the limited evidence and possible risks involved. Additionally, the use of pre-procedural antimicrobial mouthwash is considered vital before performing intraoral acupuncture.
